# Lawyer-client relationship in divorce proceedings: development and validation of a new instrument

**DOI:** 10.3389/fpsyg.2024.1444321

**Published:** 2024-08-22

**Authors:** Ana Martínez-Pampliega, Susana Cormenzana, Ma Carmen García-Garnica, Inés Pellón-Elexpuru, Laura Merino

**Affiliations:** ^1^Department of Psychology, Faculty of Health Sciences, University of Deusto, Bilbao, Spain; ^2^Department of Civil Law, Faculty of Law, University of Granada, Granada, Spain

**Keywords:** divorce, lawyer, collaborative law, assessment, parental symptomatology

## Abstract

**Introduction:**

This study is based on the paradigm of collaborative law and the current absence of instruments that evaluate the lawyer-client relationship as a function of the needs of the family system. The objective was to construct and validate an instrument, conceptualizing the lawyer-client relationship as a helping relationship.

**Method:**

Two groups of experts and 239 parents (58% mothers and 42% fathers), users of Family Visitation Centers, participated in the study. The content, construct, and criterion validity of the instrument, as well as its invariance for both parents, were analyzed.

**Results:**

The resulting 12-item instrument has been shown to have a two-dimensional structure, invariant for both parents, with high psychometric solidity.

**Discussion:**

The LCR scale seems to be a valuable and effective measure for use in a legal context, with important correlations with the parents’ psychological well-being, leading to a promising and relevant instrument for the holistic approach to the divorce process.

## 1 Introduction

It is estimated that approximately one million European families divorce annually (Eurostat, 2023). In Spain, the rate has decreased by approximately 6.7% over the last year (National Statistical Institute [INE], 2023) but still involved more than 80,000 families, of which 56.8% had underage or dependent adult offspring. This figure represents a ratio of approximately 50% of marital unions, although this figure does not consider couples’ other relational ties (Fariña et al., 2020).

Divorce is considered one of the most stressful experiences for families. Decades of cross-sectional and longitudinal studies have associated divorce with high levels of psychological distress in all the people involved, especially the children (Symoens et al., 2014; Harold and Sellers, 2018; O’Hara et al., 2019; McHale et al., 2020; Martínez-Pampliega et al., 2021), who find this impact reflected in their level of self-esteem, anxiety, depression, suicide, self-harm, substance use, behavioral problems, etc. In the same vein, D’Onofrio and Emery (2019) highlighted an increase between 1.5 and 2 in the probability of risky behaviors, living in poverty, and experiencing their own family instability. This risk can accompany them even into adulthood (Becher et al., 2018; Auersperg et al., 2019; D’Onofrio and Emery, 2019; Bhattarai et al., 2022). In this direction, Auersperg et al. (2019), through 54 studies and 506,299 participants, estimated increases between 1.35 and 1.70 in the probability of experiencing different mental health problems (suicide attempts, suicidal ideation, anxiety) or substance use in adulthood among those who experienced parental divorce in their childhood.

Despite these data, it is necessary to note that not all people suffer this impact after divorce (Perrig-Chiello et al., 2015; Martínez-Pampliega et al., 2021; Herrero et al., 2023). The differences are linked to variables surrounding the divorce experience and not so much to the divorce itself. One of the most relevant explanatory variables is the interparental conflict that takes place before and after the divorce, considering high conflict as situations of anger, unresolved grief, hostility, non-cooperative co-parenting, physical and verbal fights, and legal conflict between parents (Cummings and Davies, 2010). It is estimated that in 15 to 25 or 30% of cases, divorce is accompanied by high levels of interparental conflict and litigation (Hald et al., 2020; Arch and Fariña, 2023).

This type of conflictive divorce tends to last beyond the time required for the readaptation of the family system, chronically affecting the adjustment of the family as a system (Mills et al., 2021) and conditioning the psychological well-being of all the members involved (McHale et al., 2020; Raley and Sweeney, 2020; Martínez-Pampliega et al., 2021; Luningham et al., 2022).

### 1.1 The legal process of divorce and its impact on well-being

The procedures through which post-divorce organizational conditions are structured have been linked to the different levels of conflict between the parents and the well-being of the persons involved (Treloar, 2019; Dollar, 2020). A percentage of the cases (Hald et al., 2022; Arch and Fariña, 2023), even initially channeled through mutual agreement procedures, present a high level of conflict that translates into continuous incidents in the execution, which condition up to 90% of the resources of the Family Courts (Haddad et al., 2016). In addition, it has been highlighted that this level of conflict seems to increase and be perpetuated in contentious processes (Joyce, 2016; Francia et al., 2019; Treloar, 2019). Therefore, compared to contentious processes, collaborative Law advocates alternative conflict resolution paradigms, allowing agreements to be reached through alternative processes such as mediation (Tesler, 1999; Brophy and Crespo, 2019).

Collaborative Law is a relatively new form of ADR (Alternative Dispute Resolution) (Hla, 2018), founded in 1990 by Webb. It involves perceiving the divorce process holistically (Salava, 2014), representing not only the needs of the clients but also encouraging reflection on the needs of the ex-partner and their children.

This paradigm stems from Wexler’s work during the 1980s (Marcus, 2019), considering the courts of justice as a social agent that could have beneficial or therapeutic results for those involved. These works were the seed for the integration during the 1990s of law, ethics, and mental health (Wexler and Winick, 1996), and what is now known as Therapeutic Jurisprudence (Kawalek, 2020; Arch and Fariña, 2023).

This approach considers the legal context as an ideal terrain where different professionals can contribute to the well-being of the members who are going through the process of breakup from a systemic view of the family and targeting the well-being of the entire system (Dollar, 2020). While the role of the mental health professional is indisputable in the legal context (Fernández-Rasines, 2017), so are the roles of lawyers and judges. The paradigm of collaborative Law contributes to avoiding relitigation, which is common when there is interparental conflict (Rudd et al., 2015), so that, in turn, it indirectly contributes to reducing conflictive relationships and parental symptoms and guaranteeing the well-being and protection of the children through the exercise of positive parenting and co-parenting (McHale et al., 2020).

This practice of law requires professionals who are aware of the psychological and emotional factors involved (Dollar, 2020), with training in communication and conflict-resolution techniques (Marcus, 2019), who contribute to minimizing hostility between parents and building consensus. It is hoped that developing a good lawyer-client relationship, thereby encouraging clear and rational thinking, will contribute to the psychological adjustment of the family members.

### 1.2 The present study

The literature review has highlighted the impact of the divorce process on the psychological well-being of all the family members. In the legal context, not only the role of mental health professionals but also of legal professionals is key, although, to date, little research has focused on the lawyer-client relationship. Not only are there some relevant gaps in the conceptualization of this relationship but there are not even any evaluation instruments aimed at analyzing it, which prevents examining the explanatory mechanisms in depth. The objective of this study is to develop an assessment instrument of the lawyer-client relationship, considering it an essential step in understanding its impact on the psychological well-being of the family members.

This study will be based on conceptualizing the lawyer-client relationship as a relationship of help at the service of the functioning of the family system, connecting with the long psychological tradition of the study of the therapeutic alliance (Bordin, 1994). Specifically, it will be based on the SOATIF model (System for Observing Family Therapy Alliances) developed by Friedlander et al. (2006b), which analyzes the intensity of the alliance in the family context. This model has been strongly supported (Friedlander et al., 2018; Benítez et al., 2020) and identifies four dimensions: Commitment to the process or engagement of the people involved in the relationship to achieve the objectives; Emotional Connection or perception of a real concern and desire to help on the part of the professional; Perceived Security in the relationship, that is, ease and openness in the process; and Sharing the purpose of seeking the well-being of the family.

Therefore, the study’s objective will be to develop an instrument to evaluate the lawyer-client relationship, based on the SOATIF model, which will be validated in a group of divorced participants with a high level of conflict. Concurrent validity will be analyzed through the relationship with the parents’ psychological well-being, under the hypothesis that a better lawyer-client relationship will be related to the parents’ better psychological adjustment.

## 2 Materials and methods

### 2.1 Participants

The study’s design is instrumental and was carried out at Family Visitation Centers, that is, centers to which families with a high level of conflict are judicially referred to regulate exchanges and maintain supervised visits (De la Torre, 2018). The criteria for inclusion in the study were: (1) being divorced, separated, or in the process of being divorced; (2) having at least one minor child with the ex-spouse; (3) not living with the other parent; (4) not having a previous diagnosis of a serious psychopathology. There was no restriction criterion (procedence, sexual orientation, age…).

A total of 239 parents from 10 of Spain’s 17 autonomous communities participated in this study. Of the total number of participants, 45.6% (*n* = 109) were fathers, and 54.4% were mothers (*n* = 130), con una edad media de 42.81 (*SD* = 6.9) in the case of fathers, and 41.47 (*SD* = 7.04) in the case of mothers. The parents had separated or divorced after an average of 10.38 years of marriage (*SD* = 7.1), and in 47.9% of the cases, through a mediation process (*n* = 113) compared to 52.1% who used a contentious process (*n* = 123). In 65% of the participants (*n* = 91), the divorce had occurred at least 2 years earlier, and most of the ex-spouses had a non-existent relationship or one that was restricted to the essential aspects related to the children (87.9%, *n* = 210). With regard to their education and occupation, 58% (*n* = 138) had primary education or less, with the rest being similarly divided between those with university studies and secondary education. At the professional level, only 51.9% (*n* = 124) were active, with the rest being unemployed, retired, disabled, or on temporary leave.

At the family level, the majority (87.1%, *n* = 208) had one or two children and. Only 8.8% (*n* = 21) had joint physical custody, with the rest having joint legal custody. The relationship of the participants and ex-spouses with their children was frequent in most cases. Table 1 shows the data distributed according to the parent who responds.

**Table 1:** Sociodemographic characteristics of the sample.

	Father		Mother	
	*n*	%	*n*	%
Time since separation				
Less than 6 months	12	8,3	12	9,2
From 6 months to 1 year	3	2,8	8	6,2
1–2 years	8	7,3	6	4,6
2–3 years	13	11,9	11	8,5
More than 3 years	28	25,7	39	30
Studies				
<Primary studies	7	6,4	3	2,3
Primary studies	64	58,7	64	49,2
High school, BUP, FP	19	17,5	28	21,6
University Career	15	13,8	21	16,2
Master’s degree, doctorate	4	3,7	14	10.8
Children				
1	56	51,4	62	47,7
2	43	43	47	36,2
3	8	8	17	13,1
4+	2	2	4	1,5
Parent–child relationship				
Every day	22	20,2	103	79,2
Several days/week	22	20,2	7	5,4
Once a week	22	20,2	6	4,6
Every 2 weeks	33	30,3	11	8,5
Every month or less	10	9,1	3	2,3
Relationships with ex-partners				
Nonexistent	82	75,2	91	70
Restricted, minimal	16	14,7	21	16,2
Scarce and hostile	5	4,6	9	6,9
Fluid and polite	4	3,7	5	3,8
Variable and ambiguous	2	1,8	4	3,1
Employment status				
Unemployed	4	4,6	17	13,1
Active	67	61,5	57	43,8
Retired	3	2,8	9	6,9
Disabled	14	12,8	17	13,1
On sick leave or other	20	18,4	30	23,1
Parental custody				
JPC	12	11	9	6,9
JLC exclusive coexistence	22	20.2	103	79,2
JLC non-cohabitation	74	67,9	18	13,8
Other parent–child relationship				
Every day	71	65,1	15	11,5
Several days/week	8	7,3	30	23,3
Once a week	5	4,6	22	16,9
Every two weeks	7	6,4	37	28,5
Every month or less	18	16,5	25	19,2

BUP, Spanish high school; FP, vocational training; JPC, joint physical custody; JLC, joint legal custody.

### 2.2 Measures

Sociodemographic questionnaire: an *ad hoc* instrument was used to evaluate the variables of parents’ gender, age, education, and occupation, number of children, and their gender and age, time elapsed before and after the divorce and type of custody, and, finally, new partners, and relationships with the ex-partner and with the children.

Lawyer-Client Relationship Scale LCR-S: this instrument was developed in the present study to evaluate the relationship between the lawyer and their client. For this purpose, we adapted the instrument *Observing Family Therapy Alliances* (SOFTA-s, Alvarez et al., 2021) to the context of the lawyer-client relationship, as the starting point. The SOFTA-s comprises 12 Likert-type items (ranging from 1 = *Not at all*, to 5 = *Very much*), which are grouped into 4 dimensions of 3 items each and which include Commitment, Emotional Connection, Security, and the Sense of Sharing the family purpose in the therapeutic relationship. The instruction to answer the questionnaire in this study was: “Due to your divorce, you have probably maintained or still maintain a relationship with a lawyer. Considering that relationship, indicate to what extent you agree with the following statements.” The items were drafted so they could be accommodated to those parents who had already concluded the relationship with the lawyer, as well as to those who were still immersed in a legal process. The internal consistency of the scale was high [Cronbach’s alpha (*α*) and McDonald’s Omega (*ω*) = 0.90].

Hospital Anxiety and Depression Scale [HADS; Zigmond and Snaith, 1983; Spanish adaptation: Terol-Cantero et al. (2015)]. It is a self-report instrument of 14 items structured around two dimensions of 7 items each: Anxiety and Depression. These dimensions are used to collect symptoms linked to generalized anxiety disorder (e.g., “I get a sort of frightened feeling as if something awful is about to happen”) or depression, mainly anhedonia (e.g., “I still enjoy the things I used to enjoy”). The items are rated on a 4-point Likert scale, ranging from 0 (*Never*) to 3 (*Almost all day*), according to the frequency of symptoms during the last week. In this study, the scale showed a high internal consistency, both the total scale and the two dimensions (Total Scale: *α* = 0.91; *ω* = 0.90; Anxiety: *α* = 0.89 and *ω* = 0.90; Depression: *α* = 82 and *ω* = 0.81).

### 2.3 Procedure

The approval of the University’s Ethics Committee was obtained before developing the study (ETK-38/21–22). Then, we contacted the Family Visitation Centers of the different Autonomous Communities for the recruitment of the participants. As a first step, managers and technicians were informed of the project, initially by electronic means and/or telephone and later in person or by videoconference. Centers from 10 Spanish Autonomous Communities agreed to participate, and collaboration agreements and/or deals were signed with all of them, sometimes directly and sometimes through the Government Departments of the respective Autonomous Communities.

Once the agreements with the centers were signed, the professionals received, via telematics, informative documents about the project, the questionnaire to be answered by the users, detailed information on the informed consent, and ethical procedure required (anonymity, confidentiality, voluntariness, etc.). Finally, they were also informed about the inclusion criteria so that they could verify them in the medical history and/or in the users’ judicial referral report. A few days later, meetings were held to clarify doubts about the items and the use of the questionnaire response platform by the users. After the doubts were resolved, the professionals informed the users, who anonymously and individually decided whether to answer the questionnaires.

The data were collected online between 2020 and 2022 through a protocol of about 20 min, which participants accessed after having read the ethical conditions of the study, the registration and data custody process, and giving their consent. After their response, the participants received a check for 10 euros as an incentive, for which they had to indicate an email to that effect. The study complied with the guidelines of the Declaration of Helsinki.

### 2.4 Analysis strategies

#### 2.4.1 Adaptation and construction of the instrument

A commission made up of three researchers with training and experience in Psychology, Family Psychotherapy, and Therapeutic Alliance participated through three phases: (1) analysis of the dimensions of the SOATIF-s and adaptation of items; (2) qualitative analysis of the items by expert groups; (3) empirical study.

The first phase was based on the analysis of the SOFTA model and its self-report instrument. After the adaptation of the 12 items, content analysis was performed by two expert researchers in the field. The adequacy of the three initial dimensions was supported with 100% agreement: Commitment/involvement in the therapeutic process, Emotional connection with the therapist, and Security within the therapeutic system. These dimensions were transformed into Commitment to the legal process, Lawyer-client Emotional Connection, and Security in the lawyer-client relationship. However, the fourth dimension, sharing the family’s purpose, was inappropriate because the items referred exclusively to the relationship between the ex-spouses. For this reason, we decided to construct new items focused on the lawyer’s Family Consensus-Seeking. The initial 12 items and the 5 additional items generated an initial version of the instrument of 17 items.

In the second phase, the 17 items were subjected to a qualitative assessment by two groups of experts: on the one hand, a group of five divorced parents who responded to this version of the instrument and analyzed possible problems in the interpretation and understanding of the items, as well as their cultural relevance. On the other hand, a commission was formed by four researchers in the field of Family Evaluation and Intervention, who evaluated the wording of the items and the adequacy of the categories of responses. A minimum agreement of 75% was required.

After the assessment, questions were raised in 5 of the 17 items. Three of these were linked to the Sharing Family Purpose dimension. These items, as in the first phase, were questioned for their relevance to the direct objective of the instrument. Two more items raised questions: Item 17 (“The lawyer had training in mediation or worked in collaboration with a psychologist”), which did not reach 75% agreement and was therefore eliminated; and Item 11 (“There was a problem that I did not dare to mention to the lawyer”), which reached 75% agreement, so it remained in the pool of items. In this way, 14 items went on to the third phase, empirical analysis, which was done through processes of exploratory and confirmatory factor analysis and validity and reliability analysis. Before starting the quantitative study, the questionnaire was positively evaluated by an expert in Family Law.

#### 2.4.2 Strategies of the empirical study

Before proceeding with the exploratory factor analysis (EFA), the 14 items were initially subjected to descriptive analyses (mean, standard deviation, and asymmetry). The following exclusion criteria were established: (1) items with a mean greater than plus/minus one standard deviation of the scale measure; (2) items with a reduced standard deviation (*SD* < 0.5); (3) Pearson’s correlation coefficient between the item and the scale (<0.3); (4) an increase in the value of alpha if the item were removed (<0.2). These criteria are aimed at eliminating items with very unanimous and non-discriminatory responses, or that contribute less to the cohesion between the scale items (Morales et al., 2003; Streiner et al., 2015).

The resulting items were subjected to principal component EFA with varimax rotation. The EFA began by exploring the Kaiser–Meyer–Olkin (KMO) index and Bartlett’s sphericity test, which allowed us to identify the degree of interrelationship between the items. The reference criteria for factoring require that KMO be higher than 0.80 and that Barlett’s sphericity test be significant (*p* < 0.05). Based on the fulfillment of these assumptions, those with an eigenvalue greater than 1 were considered factors, and the items with factorial loading greater than 0.4 were accepted.

Subsequently, the results were confirmed through confirmatory factor analysis (CFA), based on structural covariance techniques and using the R program version 4.2.2 (R Core Team, 2020). Five models were examined using CFA: (a) Model 1: unifactorial model; (b) Model 2: first-order two-factor model; (c) Model 3: four first-order factors; (d) Model 4: exploratory Structural Equation Modeling (ESEM) Model: a hybrid between AFE and AFC with two first-order factors; (e) Model 5: complete bi-factor model in which items load simultaneously on one general factor and two specific factors.

Due to the absence of normal distribution of the variables, robust estimation methods were used (Maximum Likelihood Robust; MLR; Finney et al., 2016). The goodness-of-fit of the hypothesized models was assessed based on the following indices: (1) *χ*^2^ Satorra-Bentler, to analyze the divergence between variance matrices and sample covariances and that generated by the hypothesized model. This index is very sensitive to sample size (Jöreskog and Sörbom, 1993; Markland, 2007), so the relative chi-square (*χ*^2^/*df*) has also been considered; a value below 2 is strictly considered to be indicative of an acceptable fit of the model; (2) the comparative fit index (CFI), the Tucker–Lewis index (TLI), the root mean square error of approximation (RMSEA) and the standardized root mean square residual (SRMR). Excellent fit of the model was identified when the CFI and the TLI were ≥0.95, RMSEA ≤0.08 (Browne and Cudeck, 1993), and SRMR ≤0.05 (Bagozzi and Yi, 2011). In addition, the comparison of the models took into account the following three indices: Scaled *χ*^2^ Difference Test (Satorra and Bentler, 2001), Akaike’s Information Criterion (AIC), and the Bayesian Information Criterion (BIC). The difference of *χ*^2^ between the models is expected to be significant (*p* < 0.001), with lower scores on the AIC and BIC indices indicative of a better fit and lower complexity of the model.

After identifying the model with the best fit, we analyzed the loadings on the factors and the model’s power. Subsequently, the invariance of the instrument was examined, analyzing its validity according to the parent’s gender. Specifically, 7 levels of invariance were tested, which progressively increase their level of restriction: (1) configural (or equality of structure), (2) metric (equality of factor loads), (3) scalar (equality of intercepts of observed variables), (4) latvar invariance or of latent factor variances, (5) latcov invariance (equality of covariances between latent factors); (6) means (equality of means between factors); (7) residuals (equality of error variances). In addition to the AIC and BIC indices, the increase in RMSEA, CFI, and SRMR among the models was considered. An increase (△) greater than 0.01 in the CFI, 0.015 in the RMSEA, and 0.03 in the SRMR implies a significant decrease in the fit of the model (Chen, 2007). After identifying the level of invariance, the descriptive statistics (mean, standard deviation, asymmetry, and kurtosis) and internal consistency (Cronbach’s *α* and McDonald’s *Ω*) were analyzed according to the parents’ gender. Also, the existence of significant differences between parents in the instrument’s dimensions was considered using the Student’s *t*-statistic.

Finally, to analyze convergent validity, we evaluated the association between the scores of the LCR subscales and the dimensions: (1) Sharing the Purpose in the legal process (dimension not validated for use in the LCR instrument); (2) Anxiety and Depression. Pearson’s *r* correlation analysis was used, considering small values around 0.10, medium values around 0.30, and large values around 0.50 (Cohen, 1988).

## 3 Results

Table 2 integrates the descriptive analyses of each of the 14 items that comprised this second phase (mean, standard deviation, correlation, and Cronbach’s alpha). After verifying that factoring of the scale was possible (KMO = 0.927; Bartlett’s sphericity: χ91 = 3021.2; *p* ≤ 0.000, with significance <0.50), we performed the first EFA, which yielded three factors that explained 67.5% of the variance.

**Table 2:** Exploratory descriptive and factor analysis of the initial version of the LCRS.

Items	*n*	*M*	*SD*	*r*	Alpha	Factor	Eigenvalue	Explained variance	F 1	F 2	F 3
1.Lo que me planteaba el abogado/a me ayudó/me ha ayudado a solucionar nuestros problemas. The lawyer’s proposals helped me/have helped me to solve our problems.	239	2.77	1.303	0.743	0.886	1	7.380	52.714	0.854		
2.El abogado me entendió. The lawyer has understood me.	239	3.11	1.259	0.804	0.883	2	2.326	16.612	0.918		
3.Las entrevistas con el abogado/a me han servido para entender lo que necesitaba. The interviews with the lawyer helped me to understand what I needed.	239	2.96	1.334	0.812	0.882	3	1.068	7.632	0.909		
4.Siento que he estado trabajando en equipo con el abogado/a. I feel like I’ve been working as a team with the lawyer	239	2.92	1.310	0.815	0.883		Total	76.957	0.895		
5.El abogado/a ha estado haciendo todo lo posible por ayudarme. The lawyer has been doing everything they can to help me	239	3.02	1.304	0.868	0.88				0.940		
6.Me he sentido cómodo/a y relajado/a con el abogado/a. I have felt comfortable and relaxed with the lawyer.	239	3.07	1.267	0.796	0.884				0.889		
7.He entendido el sentido del proceder del abogado/a. I have understood the meaning of the lawyer’s procedure.	239	2.93	1.245	0.820	0.883				0.898		
8.El/la abogado/a es una persona importante para mí.The lawyer is an important person for me	239	2.73	1.398	0.792	0.883				0.863		
9.Ha habido algún tema del que no me he atrevido a hablar con el abogado/a. There was an issue that I did not dare to mention to the lawyer	239	0.96	1.359	−0.121	0.92						0.779
10.El abogado/a ha intentado reducir el conflicto y promover la negociación. The lawyer has tried to reduce the conflict and promote negotiation	239	2.92	1.329	0.411	0.899					0.846	
11.El abogado/a ha evitado conflictos con el objetivo de salvaguardar el bienestar de la familia. The lawyer has avoided conflicts in order to safeguard the family’s well-being.	239	3.25	1.154	0.562	0.893					0.802	
12.El abogado/a ha tenido como objetivo el mutuo acuerdo. The lawyer’s aim is to reach a mutual agreement.	238	2.72	1.438	0.383	0.901					0.842	
13.El abogado/a ha contribuido a que se tengan en cuenta los intereses de todos los miembros de la familia. The lawyer has helped to ensure that the interests of all family members are taken into account.	238	2.97	1.287	0.591	0.892					0.864	
14.El abogado/a me ha orientado para que pida un informe psicológico que favorezca mis intereses sobre los de mi pareja. The lawyer has advised me to request a psychological report that favours my interests over those of my partner.	238	1.53	1.593	0.238	0.909						0.664

Corrected alpha if the element is removed.

Two items were discarded: Item 9, which presented a mean below the criterion, a very low correlation with the other items on the scale, and an increase in internal consistency after its removal. Also, the correlation of Item 14 was below the criterion of correlation with the scale. On the other hand, the factor analysis showed that both items were integrated, constituting the third factor; however, content analysis did not validate its independent nature. As a result, both items were eliminated, and a second EFA was performed, whose data are shown in Table 3. For clarity, the items have been renumbered from 1 to 12.

**Table 3:** Exploratory factor analysis of the final version and descriptive analyses by subscale.

Item	Eigenvalue	% Variance	F1	F2	*M*	*SD*	*r*	*α*	*ω*
1	7.300	60.833	0.851		20.74	70.317	0.824	0.969	0.970
2	2.322	19.349	0.916		20.41	69.512	0.901	0.965	0.965
3		80.183	0.910		20.56	68.449	0.896	0.965	0.966
4			0.896		20.59	69.066	0.883	0.966	0.966
5			0.940		20.50	67.965	0.946	0.963	0.963
6			0.889		20.45	69.895	0.874	0.967	0.967
7			0.895		20.59	69.899	0.891	0.966	0.966
8			0.862		20.79	68.527	0.843	0.969	0.969
9				0.849	8.94	11.663	0.718	0.849	0.861
10				0.804	8.62	12.693	0.721	0.850	0.853
11				0.844	9.15	11.113	0.707	0.857	0.859
12				0.865	8.89	11.293	0.811	0.813	0.819

Corrected mean (M), variance (SD), alpha (α), McDonald’s Omega (*ω*) if the element is removed.

This second EFA (KMO = 0.931; Bartlett’s sphericity: χ66 = 2981.3, *p* ≤ 0.000), yielded two factors, which explained 80.1% of the variance. Factor loadings on both factors were greater than 0.80. The first factor comprises 8 items whose content groups the relationship between the lawyer and the client, which we called Lawyer-Client Involvement. The second factor, with only 4 items, was called Family Consensus-Seeking, as it focused on the lawyer’s support of the family’s interests as a system. Internal consistency was very high both for the two factors and the overall scale (*α* and *ω*: F1 = 0.97; F2 = 0.87; Total scale = 0.926).

Subsequently, the EFA was carried out. The goodness-of-fit indices of the five considered models are shown in Table 4. Another two models were analyzed but did not converge on a viable solution: a model with four first-order and two second-order factors and the ESEM model with four first-order factors.

**Table 4:** Comparative ANOVA of the estimated models.

	*n*	*χ*^2^_scaled_	Df	*χ*^2^/*df*	*p*	RMSEA (CI)	CFI	TLI	SRMR	AIC	BIC	*χ*^2^/*df*	Pr(>*χ*^2^)
Model 4: ESEM2	239	75,646	43	1,75	0.002	0.068 (0.042–0.092)	0.995	0.992	0.018	6731.2	6852.9		
Model 5: Bifactor	239	54,343	43	1,26	0.115	0.038 (0.000–0.066)	0.993	0.990	0.037	6693.2	6803.0	−37.961	
Model 3: 4_factores	239	62,213	48	1,29	0.082	0.042 (0.00–0.68)	0.993	0.991	0.041	6698.7	6814.9	7.345	0.196207
Model 2: Two-factor model	239	87,861	53	1,65	0.002	0.063 (0.038–0.085)	0.993	0.991	0.042	6726.9	6813.8	22.569	0.000408***
Model 1: One factor	239	360,767	54	6,68	<0.001	0.193 (0.174–0.212)	0,837	0.801	0.151	7165.8	7249.3	46.700	8.272e-12***

Model 1: unifactorial; Model 2: two first-order factors; Model 3: four first-order factors; Model 4: ESEM with two factors; Model 5: bi-factor: one general factor and two specific factors.RMSEA, root mean square error of approximation; CFI, comparative fit index; TLI, Tucker–Lewis index; SRMR, standardized root mean square residual; AIC, Akaike information criterion; BIC, Akaike information criterion.***p < 0.001.

With the exception of the unifactorial model, the other four contrasted models showed an acceptable fit, although the complete Bifactor model [*χ*^2scaled^ (df) = 54,343 (43), *p* < 0.0115, CFI = 0.993, TLI = 0.990, RMSEA = 0.038, 95% CI [0.000, 0.066], SRMR = 0.037] stood out, even ahead of the ESEM model, which presented better fit indicators in SRMR, CFI, and TLI, but whose *χ*^2^ was non-significant, and the RSMEA was slightly higher. Subsequently, the ANOVA revealed the existence of significant differences in *χ*^2^ (*p* < 0.001) of the Bifactor, the ESEM, and the 4-factor models compared to the unifactorial and bifactorial models, and identified the ESEM model as the most suitable [*χ*^2scaled^ (df) = 75,646 (43), *p* < 0.002, CFI = 0.995, TLI = 0.992, RMSEA = 0.068, 95% CI [0.042, 0.092], SRMR = 0.018]. This was supported by the analysis of the factor loadings, which are shown in Table 5.

**Table 5:** Factor loadings of the models with better fit indices.

Four first-order factors				Two-factor model			ESEM model	
F1	F2	F3	F4	Factor general	F1	F2	F1	F2
0.838				0.798	0.182		0.833	−0.004
0.933				0.885	0.274		0.921	−0.026
0.927				0.876	0.328		0.912	−0.018
	0.907			0.899	0.066		0.904	0.003
	0.979			0.983	−0.037		0.961	0.011
	0.895			0.887	0.008		0.895	−0.008
		0.922		0.900	0.066		0.892	0.042
		0.863		0.833	0.146		0.844	0.024
			0.737	0.249		0.709	−0.055	0.773
			0.807	0.397		0.699	0.107	0.758
			0.760	0.236		0.741	−0.078	0.807
			0.918	0.403		0.814	0.068	0.880

**Figure 1:**
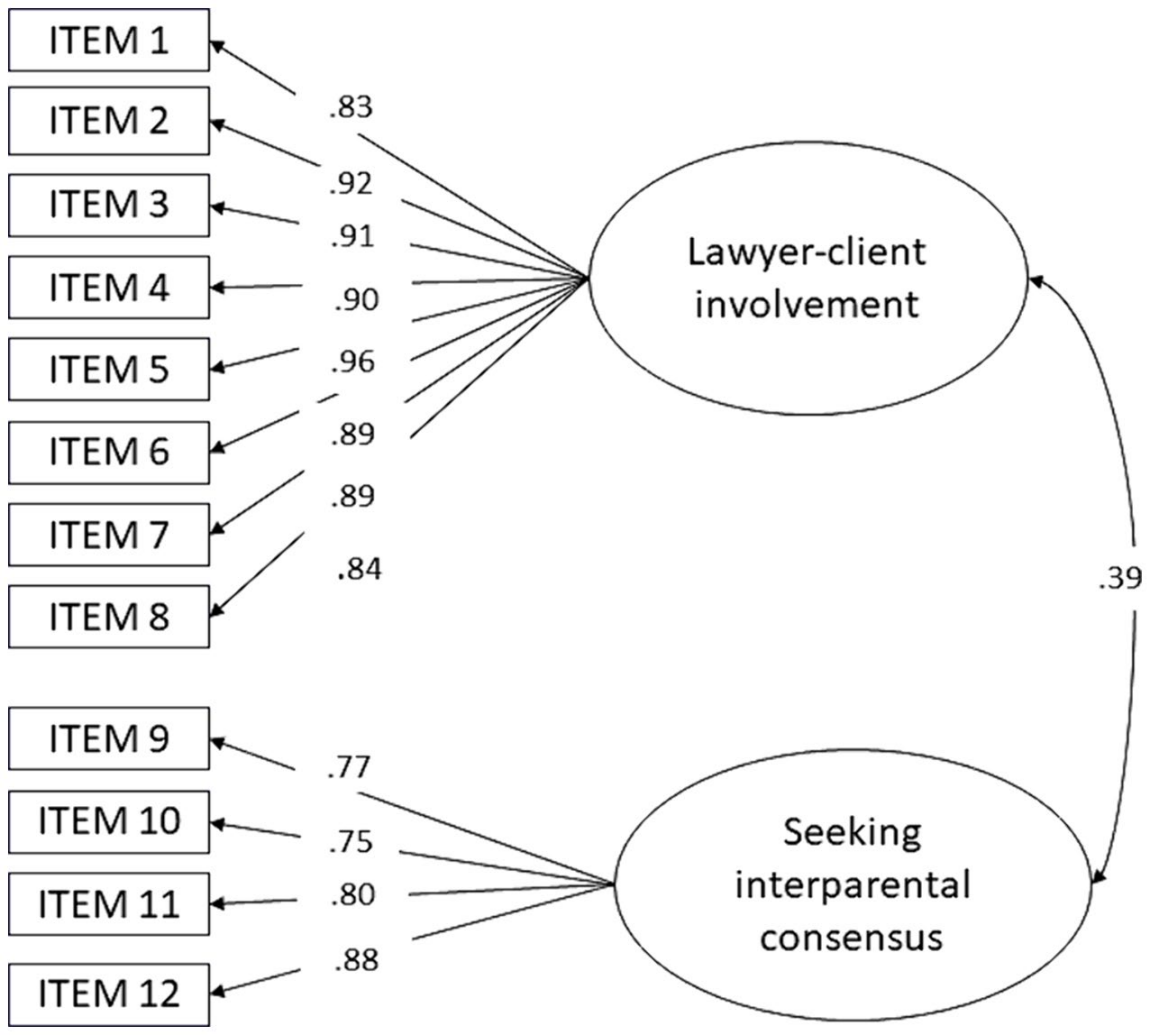
Lawyer-client relationship scale. Exploratory structural equation modeling model (ESEM) with two factors.

In the case of the ESEM model, the factorial loadings were ideal: direct loadings ≥0.75 of each item on its factor and cross-loadings ≤0.1. This was not the case with the bi-factor model. The best-fitting model is shown in Figure 1. The observed power of this ESEM model was 74% (RMSEA *p* ≥ 0.05, *n* = 239).

### 3.1 Gender invariance

The next step was to perform multigroup factor analyses to check the invariance of the instrument according to the parents’ gender. For this purpose, seven models were taken into account, with increasing restriction of equality parameters, which are shown in Table 6.

**Table 6:** Analysis of invariance according to the parent’s gender (father vs. mother).

Invariance	*χ*^2scaled^	Df scaled	*p*	RMSEA (CI)	CFI scaled	SRMR	Comparison	∆RMSEA	∆CFI	∆SRMR
Configural	119.056	86	0.011	0.057 (0.034, 0.076)	0.980	0.021				
Metric	152.232	106	0.022	0.060 (0.041, 0.078)	0.973	0.046	Configural vs. metric	−0.003	0.007	−0.025
Scalar	173.247	116	<0.001	0.064 (0.046, 0.081)	0.966	0.049	Métrica vs. scalar	−0.004	0.007	−0.003
Latvar	174.530	118	0.001	0.063	0.966	0.064	Escalar vs. Latvar	0.001	0	−0.015
Latcov	175.329	119	0.001	0.063 (0.045, 0.079)	0.967	0.073	Latvar vs. Latcov	0	−0.001	−0.009
Means	182.174	121	0.000	0.065 (0.052, 0.081)	0.964	0.083	Latcov vs. Means	−0.002	0.003	−0.01
Residuals	192.744	133	0.001	0.061 (0.045, 0.076)	0.964	0.079	Means vs. Residuals	0.004	0	0.004

Cumulative constraint models: (1) Configural model: equality of structure; (2) Metric Model: equality of factor loads; (3) Scalar Model: equality of intercepts of the observed variables; (4) Latvar model: equality of the variances of latent factors, (5) Latcov model: equality of covariances between latent factors; (6) Means Model: equality of means between factors; (7) Residuals Model: equality of error variances.RMSEA, root mean square error of approximation; CFI, comparative fit index; SRMR, standardized root mean square residual.

Although the fit of each of the models can be individually interpreted as adequate, a comparison of the overall fit was made to estimate the existence of statistically significant differences between the different levels of invariance tested. To this end, taking into account the CFI, RMSEA, and SRMR fit indices, according to the criteria of Chen (2007), Cheung and Rensvold (2002) and Rutkowski and Svetina (2014), we observed the absence of invariance in the different levels, to the extent that the increase in CFI was less than 0.01, in RMSEA less than 0.015, and in SRMR less than 0.03.

### 3.2 Descriptive statistics

Table 7 shows the *M* and *SD*, asymmetry, kurtosis, and internal consistency of the two dimensions of the instrument. The same data are indicated in the father and the mother, among whom differences were found in the Consensus-Seeking factor [*Mfathers* = 12.71, *SD* = 4.47 vs. *Mmothers* = 11.14, *SD* = 4.35, *t*(236) = 2.73, *p = 0*.007; *d* = 0.36], but not in the Involvement factor. Reliability, measured through the internal consistency of the items for the total sample and the two subsamples, obtained excellent indices, higher than 0.96 for the Involvement subscale and 0.85 for the Consensus-Seeking subscale.

**Table 7:** Descriptive statistics, internal consistency indices, and differences between fathers and mothers.

	*n*	*M*	*SD*	Skew	Kurt	*α*	*ω*
Total							
Involvement	238	23.43	9.49	−1.09	0.128	0.97	0.97
Consensus-seeking	238	11.86	4.46	−1.157	0.314	0.87	0.87
Fathers							
Involvement	109	24.23	9.29	−1.312	0.831	0.96	0.96
Consensus-seeking	109	12.71	4.47	−1.74	2.46	0.90	0.90
Mothers							
Involvement	129	22.91	9.64	−0.938	−0.272	0.96	0.96
Consensus-seeking	129	11.14	4.35	−0.767	0.046	0.85	0.85

	Father *n* = 109		Mother *n* = 130		*t*	*p*	*d*	Hedges
	*M*	*SD*	*M*	*SD*				
Involvement	24.23	9.29	22.91	9.63	1.074	0.142	0.44	0.44
Consensus-seeking	12.71	4.47	11.14	4.35	2.734	0.007	0.36	0.36

### 3.3 Concurrent validity study

Table 8 shows the correlation between LCR, Anxiety and Depression. Firstly, we note the high correlation between the two factors or subscales and the latent variable Lawyer-Client Relationship (Involvement = 0.937, *p* < 0.001; Consensus-Seeking = 0.669, *p* < 0.001). The correlation between the two dimensions was 0.36 (*p* < 0.001). Likewise, coherent relationships were observed between the LCR and its subscales, on the one hand, and the dimensions Anxiety (*r* ≥ −0.139, *p* < 0.001) and Depression (*r* ≥ −0.232, *p* < 0.001).

**Table 8:** Correlation between the LCR, anxiety, and depression.

	1	2	3	5	6
1.Lawyer-client relationship	1				
2.Involvement	0.937^**^	1			
3.Consensus-seeking	0.669^**^	0.367^**^	1		
4.Anxiety	−0.187^**^	−0.166^*^	−0.139^*^	1	
5.Depression	−0.283^**^	−0.245^**^	−0.232^**^	0.661^**^	1

*p < 0.01, **p < 0.001.

## 4 Discussion

In view of lawyers’ important role in divorce proceedings, this study aimed to develop an evaluation instrument that allows lawyers to capture their level of involvement and commitment to their clients. The result is the Lawyer-Client Relationship Scale (LCRS; see Supplementary Table S1), which, to our knowledge, is the first instrument on the relational role of the lawyer in the legal procedures of divorce. The various analyses of this study provided evidence of validity based on the test’s content, its internal structure, and the relationships with other variables, as well as other psychometric characteristics linked to the test’s reliability or invariant nature for both parents. Likewise, according to the concurrent validity hypothesis, the instrument was shown to correlate negatively and significantly with the parents’ symptomatology. The different considerations will be explained more elaborately below.

Firstly, the instrument is conceptualized as a scale that measures the relationship of help between the lawyer and the client and has the theoretical support of the SOATIF model (Friedlander et al., 2006a). Experts with different levels of involvement in the subject (professionals with training in therapeutic alliance, family relations, divorce and family law; divorced parents) supported the theoretical model and the clarity and adequacy of the items’ wording and interpretation. They also supported the cultural adaptation of the instrument to the reality of divorce through items focused on the emotional involvement between the lawyer and the client in the legal process; the lawyer’s desire to help; their ability to generate a relationship that feels secure and the ability to work from a systemic orientation, seeking the well-being of the whole family.

The different analyses related to the scale’s internal structure yielded, at the exploratory level, a two-dimensional structure explaining 80.1% of the scale’s variance, with factor loads greater than 0.80. The first factor comprises 8 items, integrating 3 items related to the lawyer’s commitment to the legal process or involvement to achieve an appropriate divorce (“I feel that I have been working as a team with the lawyer”), 3 items related to the emotional connection between the lawyer and the client, that is, the client perceives the lawyer’s appropriate concern to help and that they know how to do it (“The lawyer has been doing everything possible to help me”), 2 items focused on the security of the relationship (“I have felt comfortable and relaxed with the lawyer”), that is, the lawyer generates ease in the relationship, favoring openness and the expression of problems. This factor, taken together, is called Lawyer-Client Involvement. The second factor, with only 4 items, is called Family Consensus-Seeking, and includes the lawyer’s consideration and support for the family’s interests (“The lawyer has avoided conflicts to safeguard the family’s well-being”). The two dimensions have shown high reliability indices, analyzed through internal consistency (*α* and *ω* = 0.97, 0.87, 0.926, respectively, for the Global Scale, the lawyer-client Involvement dimension, and the Family Consensus-Seeking dimension) and mean covariance levels (*r* = 0.391, *p* = >0.001) as expected, insofar as both dimensions, although linked, analyze different aspects of the relationship, as revealed through CFA.

Through the structure analyses, it is clear that the models that best fit the data were the ESEM models, that is, hybrids between EFA and CFA, and Bifactorial analysis; in this case, the items loaded both on a general factor and specific factors. The final support was for the ESEM model and not only for empirical reasons (ANOVA), but also theoretical ones after analyzing the factor loadings (Zinbarg et al., 2006). In a bi-factor model, it is expected that, when decomposing the variance between a general factor (of greater weight) and the specific factors (of a single and lower weight, not explained by the general factor), the items will present high loadings on the general factor, but medium-low loadings on the specific factors. However, in the LCR instrument, this only occurs in the first factor, whereas in the second factor, the items present low loadings on the general factor, implying an inadequate fit of the two factors in the general factor. The lack of fit of the unifactorial model had already shown this. Compared to this model, the ESEM model allowed an adequate fit of both direct and cross-loadings, and the observed power value reached 74%, despite the small size of the sample of participants. Although this value is close to ideal (80%), reducing the model’s Beta error is necessary, which is why we need to continue developing new studies with larger samples.

Finally, concerning structure, we note two relevant aspects. On the one hand, the support for the ESEM model as opposed to the Bifactorial model raises doubts about the adequacy of working with a joint scale, to the extent that the Family Consensus-Seeking dimension is less represented on this scale, judging by the strength of the correlations (F1: *r*_F1_ = 0.937, *p* ≥ 0.001; F2: *r_F2_* = 0.669, *p* ≥ 0.001). Despite this, as will be observed later, when analyzing concurrent validity, the strength of the correlations of the global scale with other dimensions (Anxiety, Depression) is greater than that of the specific factors. All these data suggest the need for new studies to corroborate these results; meanwhile, the data argue for a cautious analysis of the results obtained in the global scale. On the other hand, we also highlight the support for the first-order four-factor model, which indirectly supports the theoretical SOATIF reference model. We can hypothesize that an increase in the number of items per dimension could more firmly support this four-dimensional structure, whereas the shorter scale developed in this study is more suitable when conceptualized from a two-dimensional approach.

Another characteristic we can affirm of this short instrument is its invariant nature, regardless of the parents’ gender, and at all levels of equality restriction. In fact, multigroup analyses reflected this to the extent that changes in CFI, RMSEA, and SRMR did not worsen significantly as a function of the level of restriction. In other words, we can affirm the existence of equality in both subsamples in terms of: (1) the identified structure, (2) the factorial loadings, (3) the measurements of the items; (4) the variances of latent factors; (5) the covariances between the latent factors, (6) the means of the factors; (7) and the error variances of the items. This equality implies that, in both parents, the scale behaves in the same way, which allows both genders to be compared more validly.

In this sense, the result was relevant when comparing the perceptions of their lawyer of the fathers and mothers in our sample. Fathers and mothers did not show differences in the assessment of the lawyer’s Involvement; they did show differences in the family Consensus-Seeking. Although the effect size is not very large, it raises questions to be addressed in future studies about this differential assessment of both parents or the lawyer’s differential actions depending on the parent’s gender. In the same vein, other variables are equally interesting, such as the lawyer’s gender, the time elapsed since the divorce, the type of custody of each parent, or the differential characteristics of the parents—training, custody, sexual orientation, etc.—, but they should be the subject of further studies.

Finally, this study provides evidence of the scale’s concurrent validity, finding important correlations between the lawyer’s role and the depressive and anxious symptomatology of divorced parents. Both factors, Involvement and Consensus-Seeking, seem inversely linked but with greater intensity on the global scale, so the better the relationship with the lawyer, the more reduced the parents’ symptoms are. Anxiety and depression are key variables in the children’s socialization and well-being, so they should be considered in post-divorce adaptation processes (Johnco et al., 2021; Ferraro and Lucier-Greer, 2022). This result is very relevant and supports the validity of working from scales that analyze the relationship of help that takes place between the lawyer and the client in a process as intensely emotional as contentious divorce. However, we cannot ignore that this is a correlational study and, therefore, we cannot venture causal hypotheses about the lawyer and the client but we can emphasize the important correlation between the two.

Therefore, the present study provides a brief 12-item scale focused on the lawyer-client involvement and the lawyer’s search for family consensus during the divorce process. This scale has been shown to be psychometrically robust due to its high levels of reliability and support for content, construct, and concurrent validity. It has also proven its validity to be used with both parents. These results support the instrument as a valid tool to understand the lawyer-client relationship in the context of highly conflictive divorce and to conceptualize the lawyer’s role in this collective, with the objective of the family’s well-being. Despite this, the study presents some limitations that should be addressed in subsequent studies. Firstly, although the sample is relevant due to its specific characteristics (users of Family Visitation Centers), it is advisable to increase the sample size and diversify the participating groups. This would allow generalizing the results to people in non-conflictive divorce proceedings, analyzing the instrument’s goodness of fit while increasing the value of its power. Secondly, the source of the data came from the divorced people themselves. Although their perception is fundamental due to its impact on interparental and parent–child relationships or their well-being, the analysis could be enriched with the perception of the lawyers themselves. Dyadic studies would make it possible to understand the different perceptions of both parties and adapt the actions to the users’ needs. This would also favor the self-monitoring of the professionals, promoting the adequacy of their interventions. Thirdly, the study only analyzed invariance according to the parent’s role, but it could be extended to include other sociodemographic variables, such as the lawyer’s gender or the lawyer-client dyad.

Another limitation is linked to the design of the study. As indicated, this is a correlational study, which implies that causal inferences cannot be made about the role of the lawyer in the study of concurrent validity, but this can be considered as a consequent line of study. In other words, with a view to future studies, this scale provides a tool with which to analyze the lawyer-client relationship from a longitudinal perspective, observing the impact of the lawyer on the divorce trajectory, while controlling the duration of the lawyer-client relationship as well as the time elapsed since its end. In addition, these studies could contribute to analyzing the explanatory mechanisms of this relationship, contemplating not only the lawyer’s characteristics (gender, education, professional trajectory, emotional regulation, communication) but also the client’s (gender, training, emotional regulation, resilience), and those of the relationship (type of custody, type of conflict, history of conflict, time elapsed since divorce), as well as examining the factors that lead lawyers to different levels of commitment and involvement.

## 5 Conclusion

From the paradigm of collaborative law, it is necessary to understand the divorce process holistically, with professionals capable of representing the needs, not only of their client, but of the entire family system. Despite the fact that this paradigm is not new and that its implication for the emotional well-being of all the members is well supported, to date, there are no instruments that identify and analyze the components of this relationship. This study has provided a brief instrument aimed at understanding the lawyer’s level of involvement and search for family consensus, conceptualizing the lawyer-client relationship as a relationship of help at the service of the functioning of the family system. This instrument has turned out to be a psychometrically robust instrument, with which to continue delving into the important role of the lawyer in the helping relationship and its impact on the chronicity of conflicts between ex-spouses. Additionally, it will make it possible to delve into the conditioning variables of the establishment of this relationship between lawyer and client, contributing to the paradigm shift so necessary in the approach to divorce processes.

## Data Availability

The datasets presented in this study can be found in online repositories. The names of the repository/repositories and accession number(s) can be found at: LCR_in Divorce Proceedings, Mendeley Data, V1, doi: 10.17632/fg7w7ztt9m.1.
