# Supplementary material for: Lawyer-client relationship in divorce proceedings: development and validation of a new instrument
Source: Front Psychol. 2024 Aug 22;15:1444321. doi: 10.3389/fpsyg.2024.1444321 (PMC11414410; doi:10.3389/fpsyg.2024.1444321)
Supplement: Supplementary file 1 [file Table_1.docx]

**Table S1**

*Lawyer-client relationship scale. Spanish version.*

Debido a su divorcio, seguramente ha mantenido o sigue manteniendo relación con algún abogado/a. Pensando en esa relación, indique en qué medida está de acuerdo con las siguientes afirmaciones / Due to your divorce, you have probably maintained or still maintain a relationship with a lawyer. Considering that relationship, indicate to what extent you agree with the following statements //

Rate the following statements:

1. Not at all
2. Not much
3. Somewhat
4. Pretty much
5. Very much

|  | 1 | 2 | 3 | 4 | 5 |
| --- | --- | --- | --- | --- | --- |
| 1. Lo que me planteaba el abogado/a me ayudó/me ha ayudado a solucionar nuestros problemas. The lawyer's proposals helped me/has helped me to solve our problems |  |  |  |  |  |
| 1. El abogado/a me ha comprendido. The lawyer has understood me |  |  |  |  |  |
| 1. Las entrevistas con el abogado/a me han servido para entender lo que necesitaba.The interviews with the lawyer helped me to understand what I needed. |  |  |  |  |  |
| 1. Siento que he estado trabajando en equipo con el abogado/a. I feel like I've been working as a team with the lawyer |  |  |  |  |  |
| 1. El abogado/a ha estado haciendo todo lo posible por ayudarme. The lawyer has been doing everything they can to help me |  |  |  |  |  |
| 1. Me he sentido cómodo/a y relajado/a con el abogado/a. I have felt comfortable and relaxed with the lawyer. |  |  |  |  |  |
| 1. He entendido el sentido del proceder del abogado/a. I have understood the meaning of the lawyer's procedure. |  |  |  |  |  |
| 1. Considero que el abogado/a se ha convertido en una persona importante para mí. I consider that the lawyer has become an important person for me. |  |  |  |  |  |
| 1. El abogado/a ha intentado reducir el conflicto y promover la negociación. The lawyer has tried to reduce the conflict and promote negotiation. |  |  |  |  |  |
| 1. El abogado/a ha evitado conflictos con el objetivo de salvaguardar el bienestar de la familia. The lawyer has avoided conflicts in order to safeguard the family's well-being. |  |  |  |  |  |
| 1. El abogado/a ha tenido como objetivo el mutuo acuerdo. The lawyer's aim is to reach a mutual agreement. |  |  |  |  |  |
| 1. El abogado/a ha contribuido a que se tengan en cuenta los intereses de todos los miembros de la familia. The lawyer has helped to ensure that the interests of all family members are taken into account. |  |  |  |  |  |

*Note:* Lawyer-Client Involvement Subscale: Items 1-8; Family Consensus-Seeking Subscale: Items 9-12.
